# Lower-dose decitabine improves clinical response compared with best supportive care in lower-risk MDS patients: a prospective, multicenter phase 2 study

**DOI:** 10.7150/jca.56207

**Published:** 2021-03-19

**Authors:** Li Ye, Chen Mei, Yanling Ren, Xinping Zhou, Liya Ma, Weilai Xu, Juying Wei, Huifang Jiang, Liming Zhang, Hui Zeng, Hongyan Tong

**Affiliations:** 1 MDS Center, Department of Hematology, the First Affiliated Hospital, Zhejiang University School of Medicine, Hangzhou 310003, Zhejiang Province, China.; 2 Key Laboratory of Hematologic Malignancies of Zhejiang Province, Hangzhou 310009, Zhejiang Province, China.; 3 Department of Hematology, Tongde Hospital of Zhejiang Province, Hangzhou 310012, Zhejiang Province, China.; 4 Department of Hematology, Zhuji People's Hospital of Zhejiang Province, Zhuji 311800, Zhejiang Province, China.; 5 Institute of Hematology, the First Hospital of Jiaxing City in Zhejiang Province, Jiaxing 314001, Zhejiang Province, China.

**Keywords:** myelodysplastic syndromes, lower risk, hypomethylating

## Abstract

**Purpose:** To explore the efficacy and safety of lower-dose decitabine in patients with lower-risk MDS, a prospective multicenter phase II study was conducted to compare decitabine with the best supportive care (BSC).

**Methods:** Patients diagnosed with lower-risk MDS from September 2013 to August 2018 were assigned to the decitabine group or the BSC group. Decitabine (12 mg/m^2^/day) was administered over 1 hour/day for 5 consecutive days in a 4-week cycle. BSC, including growth factors, transfusion, thalidomide, lenalidomide, and immunosuppressive agents were given consecutively. The endpoints included the proportion of patients who achieved overall response (OR) in the first 2 or 3 courses, event-free survival (EFS), and overall survival (OS).

**Results:** A total of recruited 82 patients were analyzed. In the decitabine group, 65.9% (27/41) achieved OR after 2 or 3 cycles of treatment, compared with 22.0% (9/41) in the BSC group (p <0.01). Besides, 44.0% (11/25) in the decitabine group became independent of RBC/Platelets transfusion, compared with 27.8% (5/18) in the BSC group. Patients with gene mutation and treated with decitabine achieved a higher OR rate, compared with those without gene mutation [72.0% (18/25) vs 11.5% (3/26), p <0.01]. There was no significant difference in the median EFS between the decitabine and BSC groups (20.6 vs 14.3 months respectively, p = 0.665). In the decitabine group, the most significant adverse events were infections of any grades or neutropenic fever (46.3%, 19/41) and one patient (4.2%) died of acute cerebral infarction within 6 weeks of treatment.

**Conclusion:** Lower-dose decitabine demonstrated promising clinical response with acceptable toxicity profiles in patients with low- and intermediate 1-risk MDS. A higher response rate to decitabine was observed in patients with mutated genes. Therefore, lower-dose decitabine can be advocated for patients with low-risk MDS and mutated genes.

## Introduction

Myelodysplastic syndromes (MDS) are characterized by peripheral cytopenias and dysplastic changes in bone marrow 1-3. Based on the International Prognostic Scoring System (IPSS) and the Revised IPSS (R-IPSS), MDS patients can be stratified according to the risk category (lower- and higher-risk). However, to enable the identification of patients with a lower-risk disease who may benefit from early treatment, Garcia-Manero et al. 4 developed a novel prognostic model (Lower-Risk PSS, LR-PSS), whereby patients with lower-risk MDS were further divided into 3 categories and demonstrated that patients in category 2 or 3 were associated with a poor prognosis if untreated. This was then independently validated by Bejar R et al. 5, which showed similar survival outcomes between lower-risk patients in category 3 and those categorized as the intermediate-2 risk in the IPSS. However, these risk-predicting models have not taken the factor of gene mutation into consideration, with several studies consistently associating gene mutation with increased levels of risk and shortened overall survival 6-8. A subgroup of lower-risk patients with poorer prognosis may benefit from early intervention.

There are several options of treatment for patients with lower-risk MDS, which include growth factors, transfusion, lenalidomide, hypomethylating agents, and immunosuppressive therapy. Decitabine represents a classic hypomethylating agent. To date, the optimal dose of decitabine in treating lower-risk patients has not been well established. Studies have examined decitabine 20 mg/m^2^/day for 5 days in lower-risk MDS patients, and revealed an overall response (OR) rate of approximately 55%, with a significantly longer OS observed among those who achieved hematologic improvement (HI) 9, 10. However, several clinical trials have also shown that a reduced dose of decitabine conferred a comparable response while minimizing treatment-related toxicities 11-15. The study by Yang AS et al. 12 has revealed that lower doses (5-20 mg/m^2^/day) of decitabine for longer periods (5-14 days) were feasible in maintaining an advantage of its demethylating properties, which was consistent with another study 13. Furthermore, decitabine 20 mg/m^2^/day for 3 days has also been shown to have a promising efficacy in lower-risk MDS patients 15. Reassuringly, the study by Jabbour E et al. 14 demonstrated an improved OR rate in patients treated with low-dose decitabine (70%, 49/70).

Therefore, we postulated that lower-dose decitabine (12 mg/m^2^/day) for consecutive 5 days might be optimal for patients with lower-risk MDS, and a phase 2 trial was conducted to compare low-dose decitabine with a single best supportive care (BSC) treatment.

## Methods

### Patients

Adult patients diagnosed with MDS between September 2013 to August 2018 and categorized as low-risk or intermediate 1-risk of the IPSS based on 2008 WHO classification 16 were eligible for this study. The patient inclusion criteria included an adequate Eastern Cooperative Oncology Group performance status (0-2), normal organ function (bilirubin <2× upper normal limit and creatinine <2× upper normal limit), and high Epo levels (more than 500 mU/mL). Patients were excluded if they met any of the following criteria: (1) secondary MDS; (2) hypocellular marrows; (3) isolated del (5q) with or without one other abnormality except -7/del (7q); (4) previously received chemotherapy, azacitidine, or decitabine before this study; (5) uncontrolled intercurrent illness; (6) active or uncontrolled infection; (7) estimated to have short survival (<3 months); (8) nursing or pregnant.

This study was approved by the Ethics Committee of the First Affiliated Hospital, Zhejiang University School of Medicine, and was registered with the Chinese Clinical Trial Registry (CHiCTR-IPR-15006454). Informed consent was obtained from all patients, conforming to the institutional guidelines and in accordance with the Declaration of Helsinki.

### Treatment regimens

Based on the clinical guidelines and patients' preferred option of the treatment regimen, they were assigned to either the decitabine group or the BSC group. The dosage of decitabine was 12 mg/m^2^/day intravenously over 1 hour on day 1 to day 5. The BSC treatments included administering growth factors, blood transfusion, thalidomide, lenalidomide, and immunosuppressive agents. When the patient failed to achieve any clinical response after 3 cycles of treatment, other treatment regimens would be instituted. On the other hand, responders could choose to undergo allogeneic hematopoietic stem cell transplantation (allo-HSCT), or consolidation courses, such as the previous regimen, or watch and wait. The duration of the consolidation treatment was until disease relapse, the progression of the disease, withdrawal of consent, intercurrent illness, or incidence of severe adverse events in the judgment of the investigators. Each course was administered every 4 weeks, as long as there were no significant myelosuppressive events, life-threatening complications such as severe infection, bleeding, or severe organ damage; otherwise, the interval could be interrupted, or extended up to 6-8 weeks. Blood transfusion, G-CSF, antimicrobial, and antifungal therapy were administered at the discretion of the physicians.

### Clinical response and toxicity

The primary outcome was the OR rate, which was assessed in the first 3 cycles according to the modified International Working Group 2006 criteria 17, including the complete remission (CR), partial response (PR), marrow CR (mCR), and hematologic improvement (HI). The secondary outcomes included overall survival (OS), event-free-survival (EFS), and transfusion independence. The OS was calculated as the number of months from the start of the therapy to the day of death or allo-HSCT, while the EFS represented the number of months from treatment initiation to disease progression, relapse, or death, whichever occurred first. The common terminology criteria (CTCAE v3.0) 18 were used for assessing toxicities.

### DNA sequencing

A target-specific next-generation sequencing (NGS) approach was used, which combined multiplex PCR-based target enrichment and library generation with ultra-deep high-throughput parallel sequencing using an Ion Proton platform 19, 20. The target genes were *SF3B1, SRSF2, U2AF1, DNMT3A, IDH1, IDH2, TET2, ASXL1, EZH2, RUNX1, ETV6, NRAS, JAK2, CBL,* and* TP53*. Mutations were annotated using multiple databases, including 1000 Genomes, COSMIC, PolyPhen-2, and dbSNP.

### Statistical analysis

All statistical analyses were performed using SPSS 22.0 software. A *P*-value of < 0.05 (2-sided) was considered statistically significant. Categorical variables were analyzed with the χ^2^ test or Fisher's exact test (for small samples), while continuous variables were analyzed with the Student's t-test. Survival curves were constructed by the Kaplan-Meier method followed by the log-rank test. Factors associated with OR, EFS, and OS were analyzed using a stepwise backward selection approach by the logistic or COX regression.

## Results

### Patient characteristics

**Figure 1:**
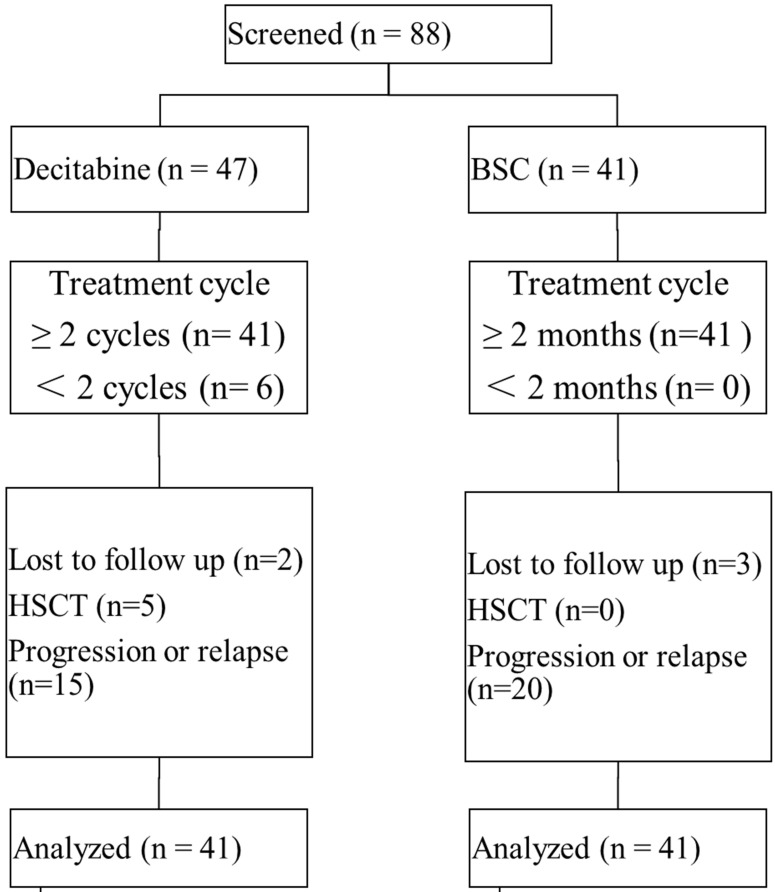
Patient disposition. Abbreviations: BSC: best supportive care; HSCT: hematopoietic stem cell transplantation.

A total of 88 patients across 4 treatment centers in China were enrolled in this study, with 82 patients available for data analyses (Figure 1). Of all patients receiving decitabine, 6 patients received only one cycle of decitabine and the treatment had to be discontinued due to the development of severe toxicity. The median number of treatment cycles in the decitabine group was 3 (IQR: 3-5 cycles). There were 5 patients undergoing allo-HSCT following the treatment with decitabine. In the BSC group, most patients received growth factors combined with thalidomide or lenalidomide (n=13), growth factors (n=13), and immunosuppressive agents (n=6). There were 85.4% (70/82) of patients classified as intermediate 1-risk MDS by IPSS, while 98.8% (81/82) of patients were in category 2 or 3 by LR-PSS. Both study groups demonstrated comparable baseline characteristics (Table 1).

NGS was performed in 75 patients (91.5%) before the commencement of treatment. At least 1 mutation was detected in 41 (62.2%) patients. The most frequently detected mutations were *TET2* (13/71, 18.3%), *U2AF1* (13/77, 16.9%), *SF3B1* (9/77, 11.7%), and *SRSF2* (7/77, 9.1%). The incidence of each gene mutation was also comparable between the two groups (Table 2).

### Treatment response

The rates of OR and CR for the entire cohort were 43.9% and 12.2% respectively. Patients treated with decitabine had significantly higher OR (65.9% vs 22.0%, p <0.01) and CR (19.5% vs 4.9%, p =0.043) rates compared with those treated with BSC (Table 3). The overall transfusion-dependent rate before treatment was 52.4% (43/82), which comprised of 25 patients in the decitabine group and 18 in the BSC group. Following treatment, 44% of patients in the decitabine group became transfusion-independent compared with 27.8% in the BSC group (p =0.278).

**Figure 2:**
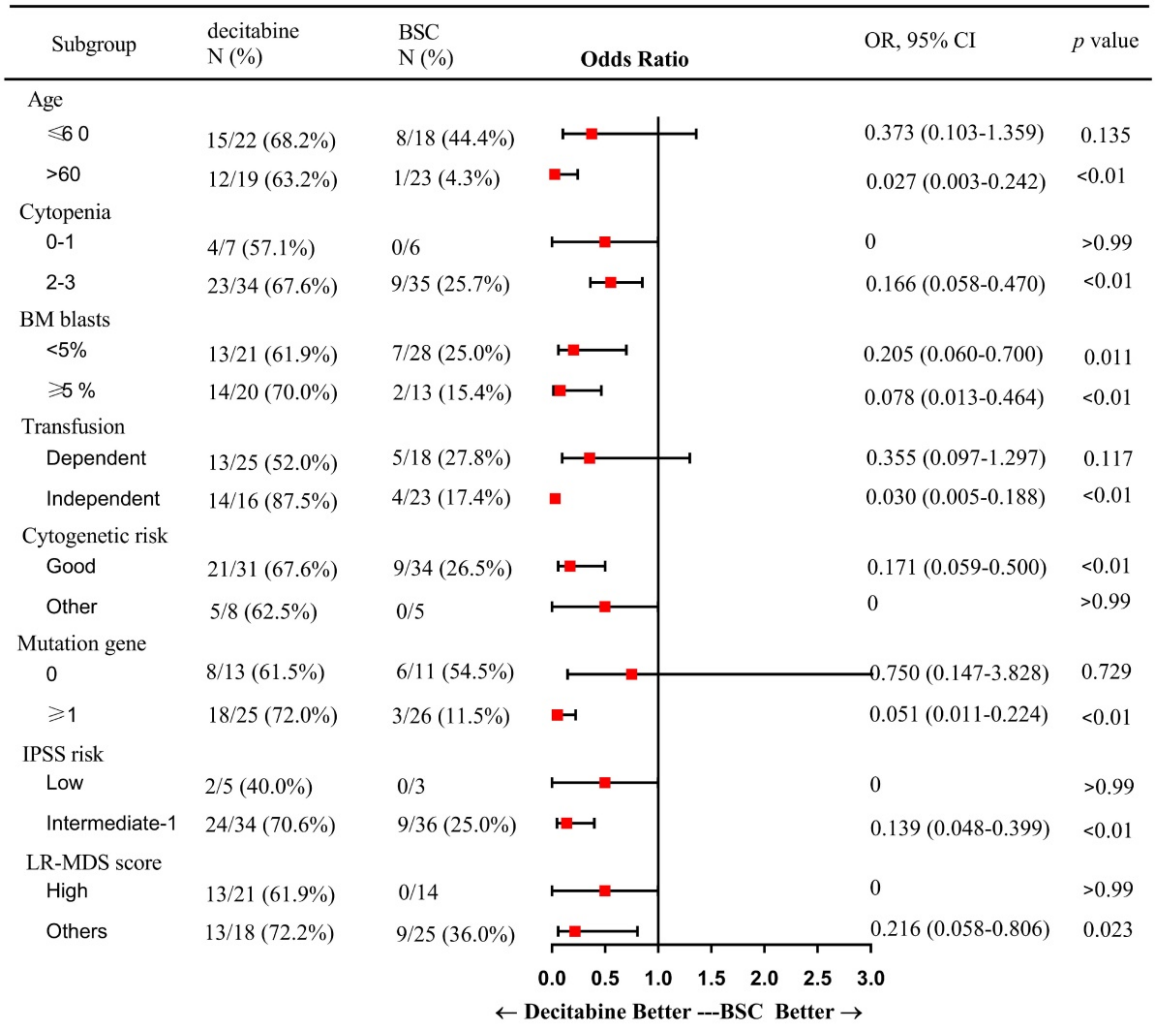
Subgroup analysis: a forest plot showing the odds ratios for overall response of various subgroups by treatment group. Abbreviations: BSC: best supportive care; OR: Odds Ratio; CI: confidence interval; BM: bone marrow; IPSS: International prognostic scoring system; LR: lower-risk.

In subgroup analysis, factors including patients of older age (> 60 years), presence of 2 or more cytopenias, good cytogenetic risk, transfusion independence, gene mutation, intermediate-1 risk disease by IPSS, and category 1 or 2 by LR-PSS were associated with a higher OR rate in the decitabine group when compared with that in the BSC group (Figure 2). Also, patients who received decitabine had a significantly higher OR rate regardless of the percentages of the bone marrow blasts.

### Patient survival

**Figure 3:**
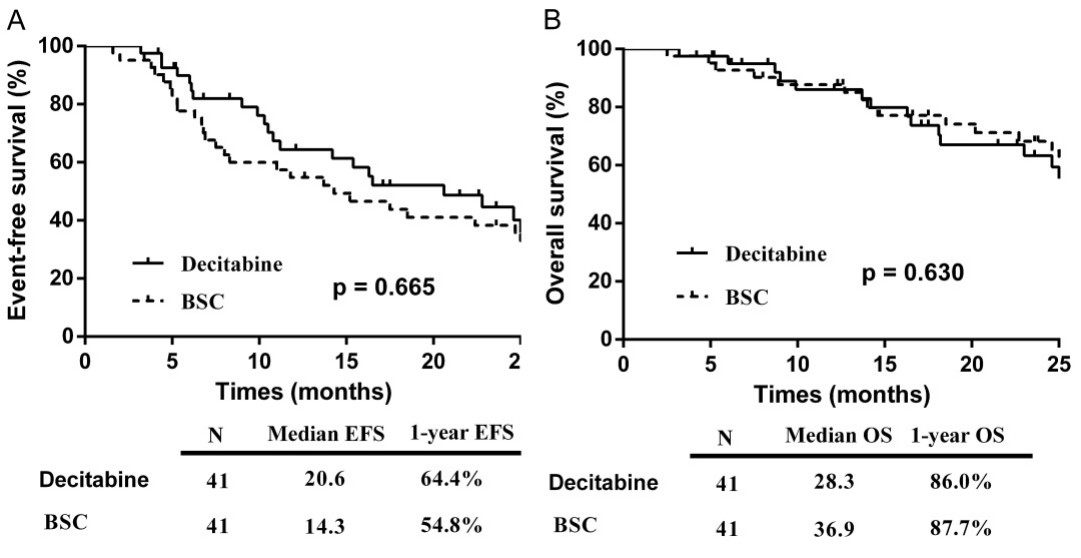
Kaplan-Meier curve: (A) EFS by treatment group; (B) OS by treatment group. Abbreviations: BSC: best supportive care; EFS: event-free survival; OS: overall survival.

The median follow-up for the entire cohort was 40.6 months (IQR: 22.6-50.4 months), with a total of 5 patients lost to follow-up (2 in the decitabine group and 3 in the BSC group). The EFS in the decitabine group (20.6 months) was longer than that in the BSC group (14.3 months), although this was not statistically significant (p = 0.665) (Figure 3A). In the univariate analyses of factors associated with EFS, patients at ≤60 years of age (p = 0.014), with < 5% bone marrow blasts (p = 0.004), achievement of OR (p =0.005), and wild-type DNMT3A (p = 0.013) affected the survival favorably. In the multivariate analysis, patients with < 5% bone marrow blasts (p=0.008) and achievement of OR (p=0.002) remained significantly associated with longer EFS.

The median OS were 28.3 and 36.9 months in the decitabine and BSC groups respectively (p = 0.630) (Figure 3B). In the univariate analysis of factors associated with OS, patients at ≤60 years of age (p <0.01), with < 5% bone marrow blasts (p = 0.017), transfusion independence (p = 0.011), wild-type DNMT3A (p <0.01), and wild-type IDH2 (p =0.033) affected the survival favorably. In the multivariate analysis, patients at ≤60 years of age (p=0.008), < 5% bone marrow blasts (p=0.024), and transfusion independence (p<0.01) remained significantly associated with longer OS.

### Toxicities

Adverse events were observed frequently in the patients treated with decitabine. In this group, grade 3/4 neutropenia was the most common (41.5%, 17/41), and 46.3% (19/41) of patients developed any grade of infection or neutropenic fever. One patient treated with decitabine died of acute cerebral infarction at week-6 of treatment.

## Discussion

In this study, we found that lower-dose decitabine was clinically effective (OR rate: 65.9%, 27/41), and patients who were transfusion independent before treatment had a higher OR rate. Our findings were consistent with a previous study by Jabbour et al. at a lower-dose decitabine regimen (20 mg/m^2^/day for 3 days) 14. Besides, Zeidan AM et al. 21 revealed that the number of transfusions in the 8 weeks before the initiation of hypomethylating agents affected the odds of achieving transfusion independence, and that patients who required more blood transfusion were associated with lower odds of transfusion independence after treatment. Also, our survival analysis suggested that decitabine treatment prolonged the EFS for another half a year when compared with BSC treatment, and a longer EFS was associated with the achievement of an OR. In a retrospective analysis by Jabbour et al. 22 concerning the treatment outcome of hypomethylating agents in 438 patients with lower-risk MDS, 77% (223/290) remained in the lower-risk disease category with the median transformation-free survival of 15 months despite treatment failure. This suggests that hypomethylating agents can delay the progression of the disease. Furthermore, a longer survival has been associated with the achievement of HI 9, especially in patients with intermediate or high-risk features.

In MDS, more than 40 recurrently mutated genes have been identified. Studies have shown that the *TET2* mutation favorably influenced the treatment response to hypomethylating agents 23, 24, while this phenomenon was not observed in the study by Idossa et al. 25. In our study, the incidence of *TET2* mutation was the most common, but mutated *TET2* was not associated with a higher OR rate when compared with the wild-type *TET2*. Nevertheless, patients in the decitabine group with at least 1 gene mutation were observed to have a higher OR rate (72.0%). Therefore, patients with the mutated gene may benefit from early intervention with a hypomethylating agent. On the other hand, patients with mutated *DNMT3A* or *IDH2* had been shown to have worse overall survival and more rapid progression to AML, compared to patients with wild-type *DNMT3A* or *IDH2* 7, 26-28. Our findings also revealed an association of *DNMT3A* or* IDH2* mutation with a shortened EFS or OS, which however were not independent prognostic factors, possibly due to the small sample size of patients harboring each of these mutations.

Several studies have shown that the incidence of adverse events is dependent on the dosage of decitabine. In the study by Lubbert M et al. 29, a high proportion of patients with intermediate- or high-risk MDS and treated with decitabine (45 mg/m^2^/day for consecutive 3 days) developed Grade 3 to 4 febrile neutropenia (25%) and infections (57%). Moreover, in lower-risk patients treated with decitabine 20 mg/m^2^/day for consecutive 3 days, Garcia-Manero G et al. 15 revealed a high rate of Grade 3 to 4 adverse events (40%) and neutropenia (28%, 12/43). However, when the treatment dose was reduced, the incidence of adverse events was also minimized, as demonstrated by Jabbour E et al. 14 that Grade 3 or more infection or neutropenia were rare when low-dose hypomethylating agents were administered. In our study, the most common toxicities related to decitabine were infection and neutropenia, in which 41.5% (17/41) developed grade 3 to 4 neutropenia and 46.3% (19/41) suffered any grades of infection. Within 3 months of treatment, one patient (4.2%) died of acute cerebral infarction at week-6. Taken into consideration of findings from our studies, our results suggested that the toxicity profiles of decitabine 12 mg/m^2^/day (for consecutive 5 days) and 20 mg/m^2^/day decitabine (for consecutive 3 days) were comparable in lower-risk MDS patients.

In conclusion, lower-dose decitabine is effective and tolerable in patients with lower-risk MDS. Patients with gene mutations may achieve a higher response rate to decitabine than best supportive care. Further research into lower-risk MDS patients with high-risk gene mutations will shed light on how those mutations may influence the clinical response to decitabine and will provide opportunities for targeted intervention.

## Figures and Tables

**Table 1:** Patient characteristics

	Decitabine (n = 41)	BSC (n = 41)	*p* value
Sex, Male/Female	25/23	43/21	0.481
Median age, (IQR; years)	59 (44-68)	61 (52-69)	0.299
Neutrophil count, (IQR; ×10^9^/L)	1.2 (0.6-1.7)	1.0 (0.7-1.9)	>0.99
Hemoglobin level, (IQR; g/L)	84 (66-109)	78 (59-93)	0.164
Platelet count, (IQR; ×10^9^/L)	43 (19-116)	49 (31-95)	0.687
BM blasts percentage, (IQR; %)	5 (2-7)	4 (2-5)	0.076
Transfusion dependence, n (%)	25 (61.0%)	18 (43.9%)	0.122
**WHO classification, n (%)**			0.085
RCUD	7 (17.1%)	3 (7.3%)	
RARS	1 (2.4%)	2 (4.9%)	
RCMD	13 (31.7%)	23 (56.1%)	
RAEB1	20 (48.8%)	13 (31.7%)	
**Baseline cytogenetic, n (%)**			0.155
del (20q)	2 (4.9%)	1 (2.4%)	
+8	3 (7.3%)	5 (12.2%)	
other	4 (9.8%)	0	
Complex (≥three abnormalities)	1 (2.4%)	0	
**IPSS risk, n (%)**			0.885
Low	5 (12.2%)	3 (7.3%)	
Intermediate-1	34 (82.9%)	36 (87.8%)	
**IPSS-R risk, n (%)**			0.258
Low	8 (19.5%)	9 (22.0%)	
Intermediate	16 (39.0%)	22 (53.7%)	
High	14 (34.1%)	8 (19.5%)	
Very high	1 (2.4%)	0	
**LR-PSS**			0.172
Category 1 (score 0-2)	0	1 (2.4%)	
Category 2 (score 3-4)	18 (43.9%)	24 (58.5%)	
Category 3 (score 5-7)	21 (51.2%)	14 (34.1%)	

Abbreviations: IQR: Inter-Quartile Range; BM: bone marrow; WHO: World Health Organization; RAEB: refractory anemia with excess blasts; IPSS: International prognostic scoring system; IPSS - R: IPSS - Revised; LR-PSS: lower-risk prognostic scoring system.

**Table 2:** Gene mutation status

Gene	Decitabine (n=41), N (%)	BSC (n=41), N (%)	*p* value
*SF3B1*	3/39 (7.7%)	6/38 (15.8%)	0.310
*SRSF2*	5/39 (12.8%)	2/38(5.3%)	0.431
*U2AF1*	5/39 (12.8%)	8/38 (21.1%)	0.335
*DNMT3A*	2/39 (5.1%)	1/38(2.6%)	>0.99
*IDH1*	1/39(2.6%)	1/38 (2.6%)	>0.99
*IDH2*	3/39 (7.7%)	0/37	0.241
*TET2*	6/37 (16.2%)	7/34 (20.6%)	0.634
*EZH2*	1/37 (2.7%)	2/34 (5.9%)	0.604
*ASXL1*	2/37 (5.4%)	4/34 (11.8%)	0.417
*RUNX1*	3/37 (8.1%)	3/34 (8.8%)	>0.99
*ETV6*	1/37 (2.7%)	0/34	>0.99
*JAK2*	1/37 (2.7%)	2/34 (5.9%)	0.604
*TP53*	2/37 (5.4%)	0/34	0.494
*NRAS*	0/34	1/34 (2.9%)	0.479
*CBL*	0/37	0/34	—

**Table 3:** Treatment response

	Decitabine (n = 41)	BSC (n = 41)	*p* value
**Response rate, N (%)**			
CR	8 (19.5%)	2 (4.9%)	0.043*
PR	0	0	—
mCR	9 (22.0%)	1 (2.4%)	0.007*
HI	14 (34.1%)	7 (17.1%)	0.077
Overall	27 (65.9%)	9 (22.0%)	<0.01*
**Transfusion response, N (%)**			
RBC	6/16 (37.5%)	5/17 (29.4%)	0.721
Platelets	7/14 (50.0%)	1/6 (6.7%)	0.325
Overall	11/25 (44.0%)	5/18 (27.8%)	0.278

Abbreviations: OR: overall response; CR: complete remission; PR: partial remission; mCR: marrow CR; HI: hematologic improvement; RBC: red blood cell. *p< 0.05.

## Abbreviations

BSC: best supportive care
IQR: Inter-Quartile Range
BM: bone marrow
WHO: World Health Organization
RAEB: refractory anemia with excess blasts
IPSS: International prognostic scoring system
IPSS-R: IPSS-Revised
LR-PSS: lower-risk PSS
OR: overall response
CR: complete remission
PR: partial remission
mCR: marrow CR
HI: hematologic improvement
RBC: red blood cell
HSCT: hematopoietic stem cell transplantation
OR: Odds Ratio (Figure 2)
CI: confidence interval
EFS: event-free survival
OS: overall survival
